# Reproductive factors and risks of biliary tract cancers and stones: a population-based study in Shanghai, China

**DOI:** 10.1038/sj.bjc.6605597

**Published:** 2010-03-09

**Authors:** G Andreotti, L Hou, Y-T Gao, L A Brinton, A Rashid, J Chen, M-C Shen, B-S Wang, T-Q Han, B-H Zhang, L C Sakoda, J F Fraumeni, A W Hsing

**Affiliations:** 1 Division of Cancer Epidemiology and Genetics, National Cancer Institute, National Institutes of Health, DHHS, Bethesda, MD, USA; 2 Department of Preventive Medicine, Northwestern University, Chicago, IL, USA; 3 Department of Epidemiology, Shanghai Cancer Institute, Shanghai, China; 4 Department of Pathology, MD Anderson Cancer Center, Houston, TX, USA; 5 Department of Epidemiology and Biostatistics, University of Pennsylvania, Philadelphia, PA, USA; 6 Shanghai Tumor Hospital, Fudan University, Shanghai, China; 7 Zhongshan Hospital, Fudan University, Shanghai, China; 8 Department of Surgery, Ruijin Hospital, Second Medical University, Shanghai, China; 9 Institute of Oriental Hepatobiliary Surgery, Second Military University, Shanghai, China; 10 Department of Epidemiology, University of Washington, Seattle, WA, USA

**Keywords:** reproductive factors, gallstones, biliary tract cancer

## Abstract

**Background:**

Parity has been linked to gallbladder cancer and gallstones, but the effects of other reproductive factors are less clear.

**Methods:**

We examined 361 incident biliary tract cancer cases, 647 biliary stone cases, and 586 healthy women in a population-based study in Shanghai.

**Results:**

The effects of parity (odds ratios, OR_⩾3 *vs* 1 child_=2.0, 95% confidence interval (CI) 0.7–5.1), younger age at first birth (OR_per 1-year decrease_=1.2, 95% CI 0.99–1.6), and older age at menarche (OR_per 1-year increase_=1.4, 95% CI 1.1–1.8) on gallbladder cancer risk were more pronounced among women with stones, but the interactions were not significant.

**Conclusion:**

Our results provide support for high parity, younger age at first birth, and late age at menarche in the development of gallbladder cancer, particularly among women with biliary stones.

Biliary tract cancers, encompassing tumours of the gallbladder, extrahepatic bile ducts, and ampulla of Vater, are uncommon but highly fatal (Hsing *et al*, 2006). Apart from a strong association with gallstones, little is known about the aetiology of these cancers (Lowenfels *et al*, 1989; Hsing *et al*, 1998, 2006). The incidence rates for gallbladder cancer and gallstones are two-fold higher in women, whereas bile duct and ampulla of Vater cancers are 50% more common in men (Hsing *et al*, 2006), suggesting that reproductive and/or hormonal factors have a role in their pathogenesis (Sama *et al*, 1990; Vitetta *et al*, 2000).

High parity is considered as a risk factor for gallstones (Thijs *et al*, 1991; Stampfer *et al*, 1992). During pregnancy, gallbladder volume increases and the flow of bile decreases, which are precursors to gallstone formation (Everson *et al*, 1982, 1991). Moreover, elevated oestrogen levels during pregnancy increase cholesterol content in the bile, contributing to biliary stasis (Braverman *et al*, 1980; Kern *et al*, 1982; Everson *et al*, 1982, 1991; Novacek, 2006). Pregnancy has also been associated with gallbladder cancer (Lambe *et al*, 1993; LaVecchia *et al*, 1993; Moerman *et al*, 1994; Tavani *et al*, 1996), but the modifying effect of gallstones and the role of other reproductive factors are not clear. We examined the risks of biliary tract cancers and stones associated with several reproductive factors among women in a population-based case–control study in Shanghai, China, where the rates of these conditions have rapidly increased in recent decades (Hsing *et al*, 2006, 1998).

## Material and methods

Details of the study have been described previously (Hsing *et al*, 2007a, 2007b, 2007c, 2007d; Andreotti *et al*, 2008; Hsing *et al*, 2008). A total of 361 women with biliary tract cancer (269 gallbladder, 92 bile duct) between 35 and 74 years of age were included. Ampulla of Vater cancer cases were excluded because of small numbers (*n*=31). In all, 70% of cancer cases were confirmed by histopathology, whereas the remaining were confirmed with medical records. We included 647 female patients with stones (511 gallstones, 136 bile duct stones) but without cancer; stone cases were confirmed with medical records. A total of 586 healthy women without cancer or stones were randomly selected from the Shanghai population. Stone status was assessed in nearly all cancer cases and controls using self-reported history and medical records.

Information on demographic, lifestyle, and reproductive factors was obtained through in-person interviews. Cancer and stone cases were interviewed within 3 weeks after diagnosis. Response rates were 95% among cases and 82% among controls. A second interview was conducted 3 months later among 5% of the subjects; the concordance of responses was 90%.

We compared gallbladder cancers with controls without a cholecystectomy (*n*=545), bile duct cancers with all controls (*n*=586), and stones with controls without stones (*n*=422). Odds ratios (ORs) and 95% confidence intervals (CIs) were calculated using unconditional logistic regression adjusted for age, education, and body mass index. Continuous variables were categorised into quartiles or tertiles on the basis of distributions within controls and those reported among Chinese women (Gao *et al*, 2000; Wernli *et al*, 2006). The likelihood ratio test was used to test for multiplicative interactions.

## Results

Of the biliary diseases examined, the strongest associations were for gallbladder cancer after adjustment for age, education, and body mass index (Table 1). Parity was positively associated with gallbladder cancer (*P*-trend=0.04). Compared with women who had one child, those having three or more children had a 2.1-fold risk (95% CI 1.0–4.2) of gallbladder cancer. Among parous women (97% of subjects), those who had their first child before 27 years of age had higher risks of gallbladder cancer relative to those who had their first child at 27 years or after, although the trend test was not significant (*P*-trend=0.15). Women who began menstruating after the age of 17 years had a 1.8-fold risk (95% CI 1.03–3.24) of gallbladder cancer, compared with those with menarche at age 13 years or younger, but the trend test was borderline significant (*P*-trend=0.05). Breastfeeding was associated with a reduced risk of bile duct cancer (OR=0.40, 95% CI 0.20–0.81), but the association became borderline significant after adjustment for parity (OR=0.90, 95% CI 0.74–1.00). Parity was not associated with biliary stones, regardless of their location in the biliary tree, age at stone diagnosis (<50 years, ⩾50 years), or duration between diagnosis and interview (<1 year, 1–4 years, 5–9 years, ⩾10 years). None of the other reproductive factors were associated with biliary stones, and there was no effect of menopausal status, age at menopause, or menstruation duration on the risk of biliary tract cancer.

The effects of parity, age at first birth, and age at menarche on gallbladder cancer risk were more pronounced among women with stones, but the interactions were not statistically significant (Table 2). Among women with stones, parity (⩾3 vs 1 child) was associated with a 1.9-fold risk (95% CI 0.74–5.09), whereas those without stones had a 1.2-fold risk (95% CI 0.25–5.84) (*P*-interaction=0.09). Among women with stones, the effect of parity was attenuated after further adjustment for age at first birth and age at menarche (OR_⩾3 *vs* 1 child_=1.47, 95% CI 0.51–4.23). Younger age at first birth (<27 *vs* ⩾27) was associated with a two-fold risk, but the estimates did not increase monotonically (*P*-trend=0.06) among those with stones; the association was weaker among those without stones (*P*-trend=0.39) (*P*-interaction=0.07). Among women with stones, the association between age at first birth and gallbladder cancer was not considerably changed when further adjusted for parity and age at menarche. Older age at menarche was significantly associated with gallbladder cancer among women with stones (OR_per 1-year increase_=1.37, 95% CI 1.06–1.77, *P*-trend=0.02), but nonsignificantly among women without stones (OR_per 1-year increase_=1.32, 95% CI 0.89–1.96, *P*-trend=0.17) (*P*-interaction=0.86). The association between age at menarche and gallbladder cancer remained significant after further adjustment for parity and age at first birth, although the estimates did not increase monotonically.

Given the strong inverse correlation between parity and age at first birth among controls (*r*=−0.66, *P*<0.0001), we examined their joint effect on gallbladder cancer among women with stones (Table 2). The risk of gallbladder cancer increased monotonically with increasing parity, regardless of age at first birth, but was highest for women having four or more children, with the first birth before the age of 23 years (OR=3.26, 95% CI 1.06–10.03). Gallbladder cancer risk also increased monotonically with increasing parity regardless of age at menarche, but the highest risk was seen for women having four or more children, with age at menarche being 16 years or older (OR=4.01, 95% CI 1.19–13.58).

## Discussion

We observed that high parity, early age at first birth, and late age at menarche contributed to gallbladder cancer risk. It is unclear why we did not find an association between parity and biliary stones, as previous studies have shown positive associations (Scragg *et al*, 1984; Basso *et al*, 1992), but some have been null (Maclure *et al*, 1989; Chen *et al*, 1999; Walcher *et al*, 2005). We lacked information on modifying factors, such as pre-pregnancy body mass index, which may have obscured an association (Basso *et al*, 1992; Lindseth and Bird-Baker, 2004; Ko *et al*, 2005). Of the 230 parous women with gallbladder cancer and biliary stones, 98% had their stones diagnosed after giving birth, and 50% >30 years after giving birth. It is likely that asymptomatic gallstones were present for many years before diagnosis, as it has been estimated that 20% of stones become symptomatic after 20 years (Diehl, 1991; Wada *et al*, 1993). The mechanism underlying the interaction between parity and stones in the development of gallbladder cancer is unclear, but may involve combined or sequential hormonal and inflammatory effects (Bartlett, 2000; Pandey and Shukla, 2000; Tazuma and Kajiyama, 2001).

Given the high correlation between parity and age at first birth, it is difficult to tease apart their individual effects. Among women with stones, a strong joint effect was evident for parity and early age at first birth. This finding is biologically plausible, as younger age at first birth may reflect higher levels and longer exposure to oestrogen and progesterone (Moerman *et al*, 1994). Although not statistically significant, there was a positive correlation between age at first birth and age of first stone diagnosis among controls, with mean ages of first stone diagnosis of 50 and 58 years for women who gave birth before the age of 21 and after 26 years, respectively.

The association between late age at menarche and gallbladder cancer is consistent with the results of some previous studies (Pandey and Shukla, 2003), but not with all (Chow *et al*, 1994; Moerman *et al*, 1994; Tavani *et al*, 1996; Zatonski *et al*, 1997). In our study, the association was independent of stones, parity, and age at first birth. It is unclear why older age at menarche was associated with higher gallbladder cancer risk, as younger age at menarche has been linked to longer exposure to oestrogen and progesterone (Moerman *et al*, 1994). As the risk estimates did not increase monotonically with increasing age at menarche, these findings should be interpreted with caution.

Breastfeeding was associated with a reduced risk of bile duct cancer, independent of stones, parity, and age at menarche, but was not associated with gallbladder cancer. Breastfeeding was related to a higher risk of gallbladder cancer in a South-American study (Strom *et al*, 1995), but not to gallbladder or bile duct cancer in a Japanese study (Kato *et al*, 1989). Although breastfeeding may have an effect through hormonal mechanisms (Bonnar *et al*, 1975; Braverman *et al*, 1980; Kern *et al*, 1982; Tazuma and Kajiyama, 2001; Novacek, 2006), our finding could be due to chance.

The null effect of menopausal status is consistent with the results of previous studies (Pandey and Shukla, 2003; Pagliarulo *et al*, 2004); however, as the majority of our population was postmenopausal (92.5% controls), we had limited statistical power to assess risk among premenopausal women. In addition, the null effect of oral contraceptive (OC) use is consistent with the results of previous studies on biliary stones (Pagliarulo *et al*, 2004; Dhiman and Chawla, 2006) and biliary tract cancers (Milne and Vessey, 1991; Chow *et al*, 1994; Moerman *et al*, 1994), but the prevalence of OC use in the population is low (20% controls) and we lacked information on duration, composition, dose, and age of use. Furthermore, few subjects received hormone replacement therapy (2.4% controls), which has been associated with gallstones (Uhler *et al*, 2000; Simon *et al*, 2001; Chen *et al*, 2006) and gallbladder cancer (Gallus *et al*, 2002; Fernandez *et al*, 2003).

In this study, selection bias was minimal because of the population-based design, high case ascertainment, and high response rates. Misclassification of cases was minimal because of the review of diagnostic data. The assessment of stone status allowed us to evaluate cancer risk while controlling for stones. The limited exposure to OCs and hormone-replacement therapy allowed us to assess reproductive factors independently of exogenous hormones. Despite being the largest population-based study on biliary tract cancers to date, we had limited statistical power to test for interactions. In addition, our findings may have limited generalisability because of the homogeneous Shanghai population.

This study revealed increased risks of gallbladder cancer associated with higher parity, younger age of first birth, and late age at menarche, primarily among those with stones, whereas there was no effect for menopausal status or OC use. The joint effects of reproductive risk factors and stones suggest the need for further study into the hormonal and inflammatory mechanisms underpinning the development of gallbladder cancer.

## Figures and Tables

**Table 1:** Odds ratios (ORs) and 95% confidence intervals (CIs) for biliary stones and biliary tract cancers in relation to reproductive factors

	**Biliary stones**					**Gallbladder cancer**					**Bile duct cancer**				
	**Controls** a	**Cases** a	**OR** b	**(95% CI)** b	***P-*value** b	**Controls** c	**Cases** c	**OR** b	**(95% CI)** b	***P-*value** b	**Controls** d	**Cases** d	**OR** b	**(95% CI)** b	***P-*value** b
Total	422	647	—	—		545	269	—	—		586	92	—	—	
															
*Parity*															
0	11	28	2.59	(0.99–6.79)		16	4	0.77	(0.20–2.99)		18	4	0.98	(0.23–4.25)	
1	67	174	1	Reference		80	26	1	Reference		81	12	1	Reference	
2	104	147	0.77	(0.50–1.21)		124	43	1.15	(0.63–2.12)		132	22	1	(0.46–2.20)	
3	70	113	1.11	(0.65–1.91)		103	52	1.60	(0.83–3.12)		111	23	1.07	(0.46–2.50)	
4	64	77	0.91	(0.50–1.66)		85	58	2.20	(1.09–4.47)		93	12	0.61	(0.23–1.65)	
5	50	54	0.80	(0.42–1.55)		67	46	2.20	(1.01–4.66)		73	7	0.43	(0.14–1.34)	
⩾6	56	53	0.79	(0.40–1.56)		70	39	1.70	(0.75–3.74)		78	12	0.68	(0.23–1.98)	
*P-*trend^e^					0.63					0.04					0.14
															
*Age at first birth (years)* f															
⩾27	112	193	1	Reference		144	45	1	Reference		150	19	1	Reference	
24–26	84	155	1.31	(0.89–1.92)		105	49	1.51	(0.92–2.48)		112	22	1.48	(0.75–2.90)	
21–23	100	129	1.13	(0.74–1.70)		128	83	1.85	(1.14–2.99)		142	25	1.22	(0.60–2.46)	
⩽20	115	141	1.10	(0.72–1.67)		151	87	1.48	(0.90–2.42)		163	22	0.91	(0.43–1.92)	
*P-*trend^e^					0.82					0.15					0.66
															
*Age at menarche (years)*															
⩽13	79	136	1	Reference		110	31	1	Reference		115	16	1	Reference	
14–15	149	244	1.05	(0.73–1.51)		193	98	1.75	(1.09–2.82)		208	38	1.26	(0.67–2.37)	
16–17	136	195	0.95	(0.65–1.39)		170	95	1.80	(1.11–2.92)		184	23	0.89	(0.45–1.78)	
⩾18	58	70	0.89	(0.55–1.43)		72	41	1.82	(1.03–3.24)		79	14	1.22	(0.55–2.72)	
*P-*trend^e^					0.52					0.05					0.95
															
*Menopause status*															
Postmenopausal	381	518	1	Reference		500	245	1	Reference		540	85	1	Reference	
Premenopausal	39	126	0.90	(0.51–1.60)	0.72	43	20	1.19	(0.53–2.66)	0.68	44	3	0.29	(0.07–1.23)	0.09
															
*Age at menopause (years)* g															
⩽46	84	139	1	Reference		107	55	1	Reference		116	20	1	Reference	
47–49	113	148	0.78	(0.53–1.14)		158	83	1	(0.65–1.53)		173	25	0.78	(0.41–1.48)	
50–52	120	160	0.81	(0.57–1.17)		152	76	0.96	(0.62–1.48)		164	28	0.95	(0.51–1.79)	
⩾53	64	71	0.63	(0.40–0.99)		83	31	0.70	(0.41–1.20)		87	12	0.78	(0.36–1.69)	
*P-*trend^e^					0.05					0.20					0.96
															
*Lifetime duration of menstruation (years)* g ^,^ h															
⩽29	138	184	1	Reference		178	107	1	Reference		195	31	1	Reference	
30–32	111	143	0.92	(0.65–1.30)		149	63	0.71	(0.48–1.04)		159	19	0.68	(0.37–1.27)	
⩾33	132	191	0.95	(0.68–1.32)		173	74	0.79	(0.54–1.16)		186	35	1.14	(0.67–1.97)	
*P-*trend^e^					0.47					0.09					0.68
															
*Ever used oral contraceptives*															
No	268	384	1	Reference		343	165	1	Reference		372	49	1	Reference	
Yes	68	110	1.09	(0.76–1.55)	0.64	84	35	0.87	(0.55–1.38)	0.54	91	19	1.62	(0.90–2.91)	0.11
															
*Breastfeeding* f															
Never	28	76	1	Reference		39	22	1	Reference		41	13	1	Reference	
Ever	383	542	0.65	(0.40–1.06)	0.08	490	242	0.73	(0.41–1.30)	0.29	527	75	0.40	(0.20–0.81)	0.01

a Biliary stone cases include gallstone and bile duct stones. Biliary stone cases compared with controls without biliary stones (*n*=422).

b Adjusted for age (continuous), education and body mass index.

c Gallbladder cancer cases compared with population controls with a gallbladder (*n*=545).

d Bile duct cancer cases compared with all population controls (*n*=586).

e Linear test of trend using continuous variable.

f Among women with at least one full-term birth (*n*=568 controls).

g Duration of menstruation=Age at menopause–age at menarche–years of pregnancy.

h Among postmenopausal women (*n*=540 controls).

**Table 2:** Odds ratios (ORs) and 95% confidence intervals (CIs) for gallbladder cancer in relation to reproductive factors by biliary stone status

	**Without biliary stones**					**With biliary stones**							
**Reproductive factors**	**Controls^a^**	**Cases^a^**	**OR^b^**	**(95% CI)^b^**	***P-*value^b^**	**Controls^a^**	**Cases^a^**	**OR^b^**	**(95% CI)^b^**	***P-*value^b^**	**OR^c^**	**(95% CI)^c^**	***P-*value^c^**
Total	422	36	—	—		123	233	—	—				
													
*Parity*													
0	11	2	—	—		5	2	—	—		—	—	
1	67	10	1	Reference		13	16	1	Reference		1	Reference	
2	104	4	—	—		20	39	2.02	(0.77–5.31)		1.77	(0.64–4.87)	
3 (⩾3 for no stones)	240	20	1.21	(0.25–5.84)		33	43	1.35	(0.52–3.49)		1.23	(0.44–3.43)	
4	—	—	—	—		21	53	2.89	(1.02–8.23)		2.55	(0.81–8.05)	
5	—	—	—	—		17	42	2.86	(0.95–8.62)		2.38	(0.69–8.15)	
⩾6	—	—	—	—		14	37	2.99	(0.93–9.65)		2.43	(0.65–9.07)	
*P-*trend^d^			—	—	0.57			—	—	0.03	—	—	0.16
*P-*interaction										0.09			
													
*Age at first birth (years)* e													
⩾27	112	9	1	Reference		32	36	1	Reference		1	Reference	
24–26	84	10	1.83	(0.66–5.06)		21	39	1.77	(0.84–3.71)		1.74	(0.81–3.76)	
21–23	100	9	1.21	(0.39–3.82)		28	74	2.56	(1.28–5.13)		2.36	(1.12–4.99)	
⩽20	115	6	0.59	(0.16–2.22)		36	81	1.96	(0.97–3.95)		1.86	(0.84–4.15)	
*P-*trend^d^					0.39					0.06			0.13
*P-*interaction										0.07			
													
*Age at menarche (years)*													
⩽13	79	5	1	Reference		31	26	1	Reference		1	Reference	
14–15	149	11	1.10	(0.35–3.43)		44	87	2.29	(1.20–4.40)		2.20	(1.12–4.29)	
16–17	136	12	1.32	(0.43–4.03)		34	83	2.71	(1.37–5.33)		2.60	(1.29–5.24)	
⩾18	58	8	2.33	(0.67–8.14)		14	33	2.61	(1.12–6.09)		2.28	(0.96–5.39)	
*P-*trend^d^					0.17					0.02			0.04
*P-*interaction										0.86			
													
*Parity and age at first birth (years)*													
0–1 and ⩾24	—	—	—	—		10	14	1	Reference		—	—	
2–3 and ⩾24	—	—	—	—		31	43	1.47	(0.52–4.16)		—	—	
⩾4 and ⩾24	—	—	—	—		12	18	1.86	(0.49–6.94)		—	—	
0–1 and ⩽23	—	—	—	—		2	2	—	—				
2–3 and ⩽23	—	—	—	—		22	39	1.77	(0.59–5.20)		—	—	
⩾4 and ⩽23	—	—	—	—		40	114	3.26	(1.06–10.03)		—	—	
													
*Parity and age at menarche (years)*													
0–1 and ⩽15	—	—	—	—		12	12	1	Reference		—	—	
2–3 and ⩽15	—	—	—	—		37	44	1.48	(0.49–4.40)		—	—	
⩾4 and ⩽15	—	—	—	—		26	57	3.19	(0.95–10.69)		—	—	
0–1 and ⩾16	—	—	—	—		6	6	1.12	(0.21–6.09)		—	—	
2–3 and ⩾16	—	—	—	—		16	36	2.56	(0.79–8.21)		—	—	
⩾4 and ⩾16	—	—	—	—		26	74	4.01	(1.19–13.58)		—	—	

a Gallbladder cancer cases compared with population controls with a gallbladder (*n*=545).

b Adjusted for age (continuous), education and body mass index.

c Further adjusted for age first birth (continuous), age menarche (continuous) and parity; each factor is not self-adjusted.

d Linear test of trend using continuous variable.

e Among women with at least one full-term birth (*n*=568 controls).
